# Physiological and Transcriptomic Analyses Reveal Regulatory Mechanisms of Adventitious Root Formation in In Vitro Culture of *Cinnamomum camphora*

**DOI:** 10.3390/ijms26157264

**Published:** 2025-07-27

**Authors:** Yuntong Zhang, Ting Zhang, Yongjie Zheng, Jun Wang, Chenglin Luo, Yuhua Li, Xinliang Liu

**Affiliations:** 1 State Key Laboratory of Tree Genetics and Breeding, Beijing Forestry University, Beijing 100083, China; canon534534@163.com (Y.Z.); wangjun@bjfu.edu.cn (J.W.); 2 Camphor Engineering and Technology Research Centre of National Forestry and Grassland Administration, Jiangxi Academy of Forestry, Nanchang 330032, China; zhangtycx@163.com (T.Z.); zyj_bio2015@163.com (Y.Z.); l18359088807@163.com (C.L.); lyh8230110048@njfu.edu.cn (Y.L.)

**Keywords:** morphogenesis, oxidoreductases, endogenous hormones, RNA-seq, temporal dynamics

## Abstract

*Cinnamomum camphora* is an ecologically and economically significant species, highly valued for its essential oil production and environmental benefits. Although a tissue culture system has been established for *C. camphora*, large-scale propagation remains limited due to the inconsistent formation of adventitious roots (ARs). This study investigated AR formation from callus tissue, focusing on associated physiological changes and gene expression dynamics. During AR induction, contents of soluble sugars and proteins decreased, alongside reduced activities of antioxidant enzymes, including superoxide dismutase (SOD), peroxidase (POD), and polyphenol oxidase (PPO). Levels of indole-3-acetic acid (IAA) and abscisic acid (ABA) decreased significantly throughout AR formation. Zeatin riboside (ZR) levels initially declined and then rose, whereas gibberellic acid (GA) levels displayed the opposite trend. Comparative transcriptomic and temporal expression analyses identified differentially expressed genes (DEGs), which were grouped into four distinct expression patterns. KEGG pathway enrichment indicated that 67 DEGs are involved in plant hormone signaling pathways and that 38 DEGs are involved in the starch and sucrose metabolism pathway. Additionally, protein–protein interaction network (PPI) analysis revealed ten key regulatory genes, which are mainly involved in auxin, cytokinin, GA, ABA, and ethylene signaling pathways. The reliability of the transcriptome data was further validated by quantitative real-time PCR. Overall, this study provides new insights into the physiological and molecular mechanisms underlying AR formation in *C. camphora* and offers valuable guidance for optimizing tissue culture systems.

## 1. Introduction

*Cinnamomum camphora*, a valuable evergreen species widely distributed across East Asia, is highly regarded for its ecological resilience and economic value [1]. Its remarkable adaptability and resistance to abiotic stress make it a key species in ecological restoration efforts. The fine-grained, durable wood of *C. camphora* is sought after for high-end furniture production, while its roots, stems, and leaves—rich in essential oils—serve as important raw materials for the chemical, spice, food, and pharmaceutical industries [2]. Propagation of *C. camphora* is achieved through both sexual reproduction via seeds and clonal propagation techniques [3]. However, seed-based propagation is limited by low germination rates and high genetic variability, while cutting-based methods suffer from inconsistent rooting, low survival rates, and strong dependence on seasonal and maternal plant conditions. These limitations significantly hinder the efficient utilization and conservation of *C. camphora* germplasm resources. Tissue culture techniques offer a promising solution by enabling large-scale, rapid clonal propagation that preserves the elite traits of the donor plant and are not constrained by seasonality or source material [4].

Plant tissue culture technology is a sophisticated and efficient method of asexual propagation, widely utilized in rapid seedling production, generation of virus-free plantlets, plant breeding, and large-scale commercial cultivation. Among the critical stages in tissue culture, adventitious root (AR) formation plays a pivotal role, as it establishes a functional root system that enhances water and nutrient absorption, thereby significantly improving transplantation success rates [5]. In tissue culture, ARs can be generated through two distinct regeneration pathways: directly from excised explants or indirectly via callus formation [6,7]. The regulatory mechanisms underlying AR development are influenced by a complex interplay of internal and external factors, including the physiological state of the explants, the composition of the basal medium, the type and concentration of exogenous hormones, as well as the concentrations of sucrose, agar, activated carbon (AC), and the pH of the culture medium. Previous studies have shown that half-strength Murashige and Skoog (1/2 MS) medium, with reduced macronutrient content, is more conducive to AR induction [8]. Additionally, the process of AR formation demands substantial metabolic energy due to rapid cell division and heightened biosynthetic activity, necessitating considerable carbohydrate consumption—primarily in the form of sugars [9]. Moreover, AC has been widely employed in AR induction protocols for its ability to absorb inhibitory substances and simulate a low-light environment favorable to root development [10]. In tissue culture, the induction of AR is often accompanied by oxidative stress, characterized by the accumulation of reactive oxygen species (ROS) [11]. Oxidoreductases such as SOD, POD, and PPO are actively involved in modulating ROS levels, thereby creating a cellular environment conductive to cell dedifferentiation, reprogramming, and root primordia initiation [12].

The addition of appropriate concentrations of plant hormones to the culture medium significantly promotes rooting, as AR formation is closely associated with dynamic changes in endogenous hormone levels. Among these, auxin plays a central regulatory role in AR induction. Auxin directly or indirectly regulates root primordium initiation and promotes AR elongation [13]. Due to its higher stability compared to natural indole-3-acetic acid (IAA), the synthetic auxin 1-naphthaleneacetic acid (NAA) is commonly used to enhance AR formation in tissue culture systems [14,15]. Exogenous application of NAA was found to be highly effective in promoting adventitious root development in tissue-cultured plantlets of *C*. *camphora*, achieving a rooting rate of over 90% [16,17]. Exogenous auxin affects the balance of endogenous auxin. Previous studies have demonstrated that NAA reduced the levels of endogenous IAA in the cuttings of *Camellia sinensis*, *Populus tomentosa*, and *Populus davidiana* [18,19]. Exogenous application of NAA modulated peach fruit growth and development by activating the auxin signaling pathway and inducing the expression of auxin-responsive genes [20]. NAA primarily enters cells via passive diffusion, modulates the auxin concentration gradient, and indirectly influences its polar transport [21]. By bypassing AUX1/LAX-mediated uptake, *AUX1* belongs to the four-member *AUX1/LAX* family in *Arabidopsis*, encoding proteins homologous to amino acid transporters, facilitating auxin influx into cells [22]. Via AUX1/LAX-mediated uptake, auxin binds to TIR1/AFB receptors, triggering the ubiquitin-mediated degradation of AUX/IAA repressor proteins [23]. This process releases auxin response factors (ARFs), which then activate downstream gene expression [24]. In particular, *ARF7* and *ARF19* upregulate the expression of *LATERAL ORGAN BOUNDARIES DOMAIN* (*LBD*) genes such as *LBD16* and *LBD29*, thereby initiating root primordium formation [25]. *LBD16* further induces the expression of *WUSCHEL-RELATED HOMEOBOX 5* (*WOX5*), which maintains stem cell identity at the root tip [26]. In *Populus*, *WOX5* has been shown to play a key role in regulating root apical meristem development [27,28]. During the AR elongation phase, auxin promotes root extension by inducing the expression of *Extensin1* (*EXT1*), a gene associated with cell wall loosening [29]. In contrast, cytokinin acts as a negative regulator of AR formation, suppressing root primordium differentiation in cucumber hypocotyls and *Populus tremula* cuttings [30,31]. High concentrations of auxin activate ARFs, which inhibit the expression of *IPT3* and *IPT5*, thereby reducing cytokinin biosynthesis and alleviating its inhibitory effects [32]. ABA is generally considered a rooting inhibitor in many wood plants [33]. The PYR/PYL–PP2C–SnRK2 module is a key ABA signaling pathway [34]. Upon ABA binding, the PYR/PYL receptors inhibit PP2C, releasing SnRK2 to activate downstream transcription factors involved in developmental regulation [35].

Although tissue culture techniques have successfully induced rooting in *C. camphora* plantlets, transcriptomic analysis remains essential for understanding the underlying regulatory mechanisms. Root formation is a complex, multi-stage process governed by the coordinated action of multiple phytohormones, primarily auxin and cytokinin. However, current tissue culture protocols are largely based on empirical optimization and lack a comprehensive molecular understanding. In this study, tissue-cultured seedlings induced on a rooting medium were used as experimental materials. Key physiological parameters—including soluble sugar, soluble protein, malondialdehyde (MDA), and the activities of SOD, POD, and PPO—were quantified. Additionally, dynamic changes in phytohormones such as IAA, ZR, GA, and ABA were measured at different developmental stages. To elucidate the molecular events associated with AR formation, transcriptome sequencing was performed to identify differentially expressed genes (DEGs) across distinct stages, including callus induction and AR emergence. Temporal expression trends of DEGs were analyzed using Mfuzz, a fuzzy clustering algorithm capable of grouping genes with similar dynamic expression patterns over time. This method effectively captures the stage-specific transcriptional profiles essential for understanding developmental transitions [36]. By integrating physiological measurements with transcriptomic data, this study aims to uncover the molecular basis of AR formation in *C. camphora* under tissue culture conditions.

## 2. Results

### 2.1. Morphological and Anatomical Changes During AR Formation

**Figure 1:**
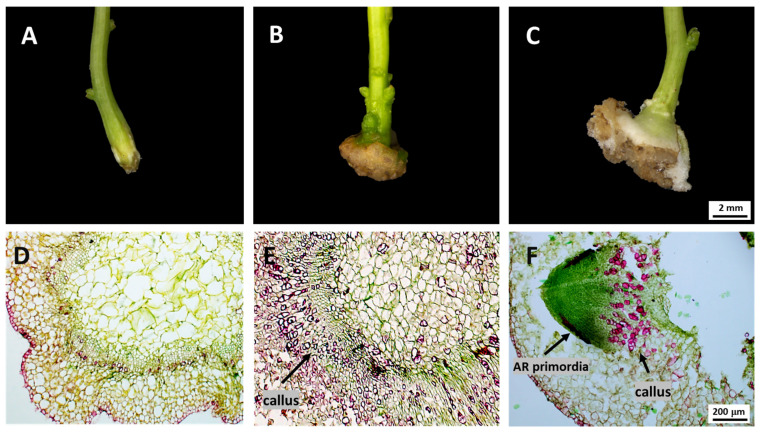
The morphological and anatomical change of the basal region of the cuttings during AR formation. (**A**–**C**) Morphological observations. (**D**,**E**) Anatomical observations. (**A**,**D**) ZM1. Callus induction. (**B**,**E**) ZM2. Callus formation. (**C**,**F**) ZM3. AR emergence.

Morphological observations were conducted to characterize the process of AR formation in *C. camphora* under tissue culture conditions. Following transfer to the rooting medium, the basal region of the explants exhibited noticeable swelling (Figure 1A), followed by the development of callus tissue (Figure 1B). As the callus expanded, small white protrusions resembling buds appeared on its surface (Figure 1C), which subsequently elongated and gave rise to lateral roots. These observations indicate that *C. camphora* undergoes a callus-mediated pathway of AR formation in vitro. Microscopic examination of anatomical sections at different developmental stages further elucidated the progression of AR formation (Figure 1D–F). At stage ZM1, no signs of root primordia were observed at the base of the stem segments (Figure 1D). By stage ZM2, a distinct aggregation of small, densely arranged cells was visible near the cambial region at the stem base, indicative of active callus formation (Figure 1E). At stage ZM3, a dome-shaped root primordium was observed breaking through the callus surface, marking the onset of AR emergence (Figure 1F). Subsequently, the emerging root structure continued to elongate and differentiate, reflecting the progression of AR development.

### 2.2. Changes in Nutrient Content and Enzyme Activities During AR Formation

**Figure 2:**
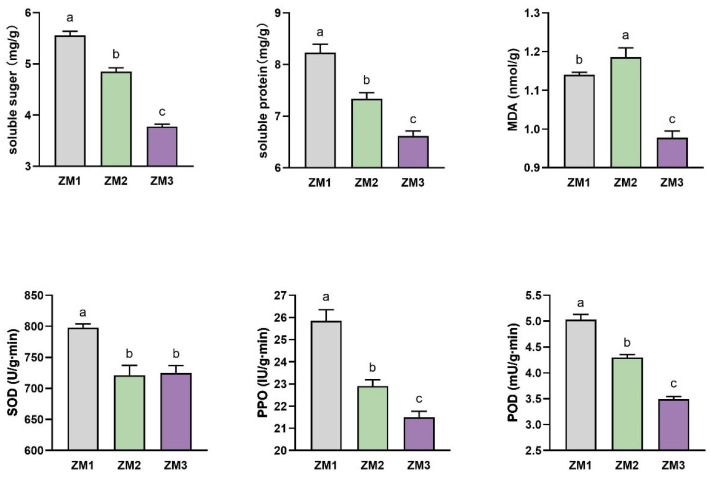
Changes in nutrient content and enzyme activity in different stages. ZM1, ZM2, and ZM3 represent callus induction, callus formation, and AR emergence stages, respectively. Note: *n* = 6 for each treatment group. Different lowercase letters indicate significant differences (*p* < 0.05).

During AR formation in *C. camphora* tissue cultures, both soluble sugar and soluble protein contents showed a continuous and significant decline (Figure 2). Oxidative stress markers and antioxidant enzyme activities also exhibited dynamic changes throughout AR formation. MDA content increased during the early stage of root primordium development, suggesting transient accumulation of reactive oxygen species (ROS) and lipid peroxidation. However, MDA levels subsequently declined upon root emergence, indicating alleviation of oxidative stress. SOD activity showed a sharp increase during the callus induction and early rooting stages, catalyzing the conversion of superoxide radicals (O_2_^−^) into hydrogen peroxide (H_2_O_2_) to mitigate oxidative damage. SOD activity then stabilized during root elongation, contributing to the maintenance of redox homeostasis. In contrast, the activities of POD and PPO consistently decreased throughout the rooting process, suggesting a reduced role in ROS production and phenolic oxidation during the transition from cell dedifferentiation to organized root development (Figure 2).

### 2.3. Changes in Endogenous Hormone Levels During AR Formation

**Figure 3:**
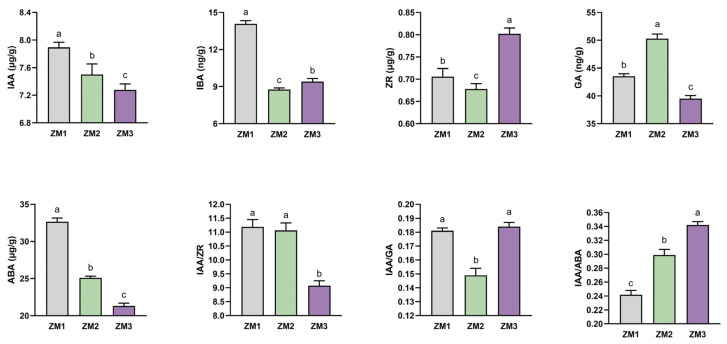
Changes in endogenous hormone levels during AR formation. ZM1, ZM2, and ZM3 represent callus induction, callus formation, and AR emergence stages, respectively. Note: *n* = 6 for each treatment group. Different lowercase letters indicate significant differences (*p* < 0.05).

The endogenous concentrations of IAA, ABA, GA, IBA, and ZR exhibited distinct and dynamic changes during the process of AR formation. IAA levels declined markedly throughout all stages. GA levels displayed a bell-shaped trend: they initially increased during callus induction, presumably to promote cell division and callus expansion, and then significantly decreased as root formation progressed. ABA content showed a steady and substantial decline over the entire course of AR development. ZR content gradually decreased from ZM1 to ZM2, reflecting the antagonistic interaction between cytokinin and auxin during callus initiation. However, a sharp increase in ZR was observed during AR emergence (ZM3). In terms of hormone ratios, the IAA/ZR ratio exhibited minimal change during callus formation but declined during AR emergence. The IAA/GA ratio showed an inverse pattern relative to GA levels. Notably, the IAA/ABA ratio increased progressively throughout AR formation (Figure 3).

### 2.4. Transcriptomic Analysis of Critical Stages of AR Formation

Based on morphological, anatomical, and endogenous hormone profiling, stem base samples from three critical time points—ZM1, ZM2, and ZM3—were selected for transcriptome sequencing (RNA-seq), with three biological replicates per stage. Biological replicates exhibited high correlations (r > 0.9), confirming the consistency and reliability of sample processing (Figure S1). After stringent quality filtering and control, each of the nine libraries yielded no fewer than 40 million clean reads. The Q20 and Q30 values exceeded 98.59% and 94.89%, respectively, indicating high sequencing accuracy. The GC content ranged from 45.82% to 46.12%, reflecting stable base composition across libraries. While 4.98–6.15% of reads were mapped to multiple locations, a high percentage (91.25–92.66%) were uniquely mapped.

**Figure 4:**
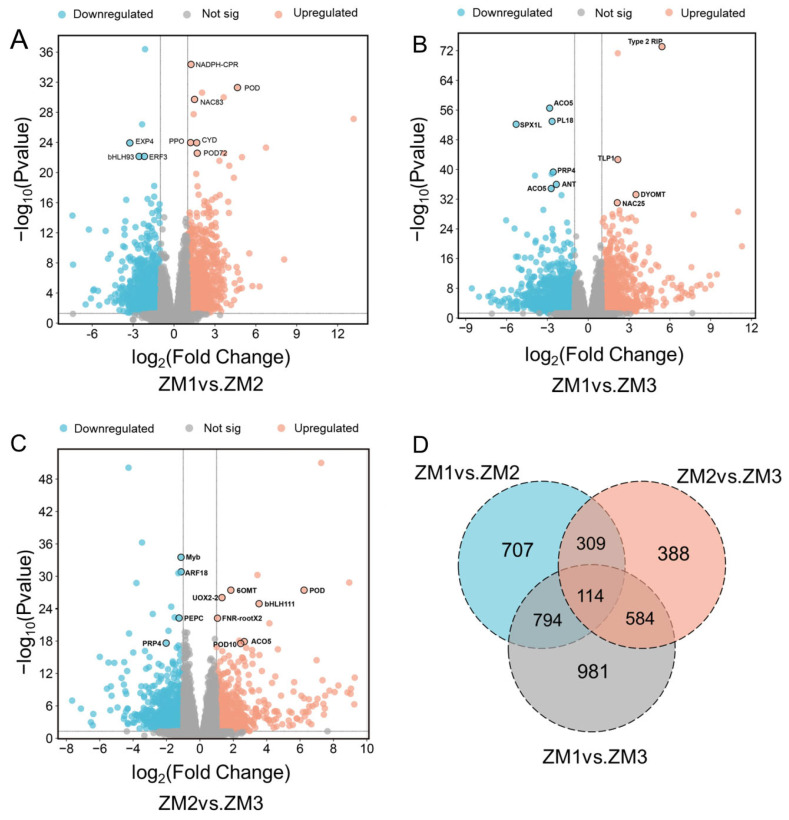
Volcano plots and Venn diagram of DEGs. In the volcano plots, each point represents a gene. (**A**) Volcano plot of ZM1 vs. ZM2. (**B**) Volcano plot of ZM1 vs. ZM3. (**C**) Volcano plot of ZM2 vs. ZM3. (**D**) Venn diagram of DEGs among the three comparisons. The *x*-axis shows the log2 fold change, which indicates the log2 value of the fold change in expression levels of a gene between two samples. The *y*-axis represents either the *p*-value or the false discovery rate (padj), with the *y*-value being the negative log10-transformed value of the *p*-value or padj. ZM1, ZM2, and ZM3 represent callus induction, callus formation, and AR emergence stages, respectively.

DEGs were identified using thresholds of *p* < 0.05 and |log_2_(fold change)| > 1 (Figure S2). In the ZM1 vs. ZM2 comparison, 970 genes were significantly upregulated and 954 were downregulated (Figure 4A). In the ZM2 vs. ZM3 comparison, fewer DEGs were detected, with 549 upregulated and 846 downregulated genes (Figure 4B). The largest number of DEGs was observed between ZM1 and ZM3, comprising 955 upregulated and 1518 downregulated genes (Figure 4C).

### 2.5. Temporal Dynamics of DEGs

AR formation in *C. camphora* comprises three distinct stages: callus induction, callus formation, and AR emergence. To elucidate the temporal regulatory patterns of gene expression throughout this process, we employed fuzzy c-means clustering using the Mfuzz package (version 2.68.0) in R (version 4.5.0). Based on the elbow method, the optimal cluster number was selected at the point where the decrease in within-cluster variability began to plateau (Figure S3). A total of 3877 DEGs were classified into six clusters (C1–C6), which were subsequently grouped into four major temporal expression patterns: Group 1 (C1 and C6), characterized by an initial increase in expression followed by a decline; Group 2 (C5), which displayed a continuous downregulation across all stages; Group 3 (C2), which showed an initial decrease followed by a later upregulation; Group 4 (C3 and C4), which exhibited a steady increase in expression over time.

**Figure 5:**
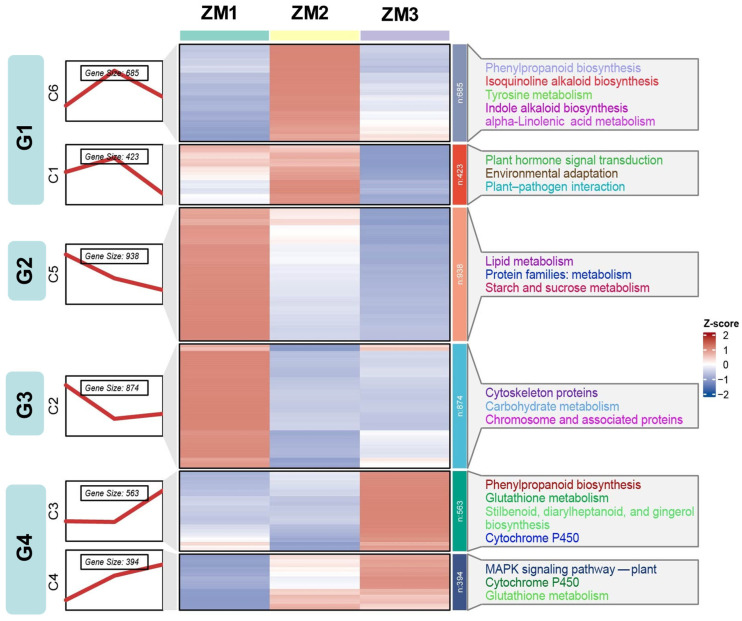
MFUZZ cluster and KEGG enrichment analysis of DEGs involving three point comparison groups. The colors represent the relative gene expression values after normalization adjustments. ZM1, ZM2, and ZM3 correspond to callus induction, callus formation, and AR emergence stages, respectively. The red and blue colors refer to up- and downregulation.

KEGG pathway enrichment analysis revealed distinct biological functions associated with each expression pattern. Group 1 was significantly enriched in pathways related to signal transduction and secondary metabolite biosynthesis, including plant hormone signaling and phenylpropanoid metabolism. Group 2 was primarily involved in lipid metabolism, starch and sucrose metabolism, and metabolic protein families. Group 3 was enriched for genes associated with cytoskeletal components, carbohydrate metabolism, and chromosome-related proteins. Group 4 was linked to phenylpropanoid biosynthesis, glutathione metabolism, cytochrome P450 enzyme systems, and MAPK signaling pathways (Figure 5). These results provide a comprehensive overview of the dynamic transcriptional landscape underlying AR development in *C. camphora*, offering critical insights into the temporal coordination of gene regulatory networks during in vitro root formation.

### 2.6. GO and KEGG Enrichment Analysis of DEGs

**Figure 6:**
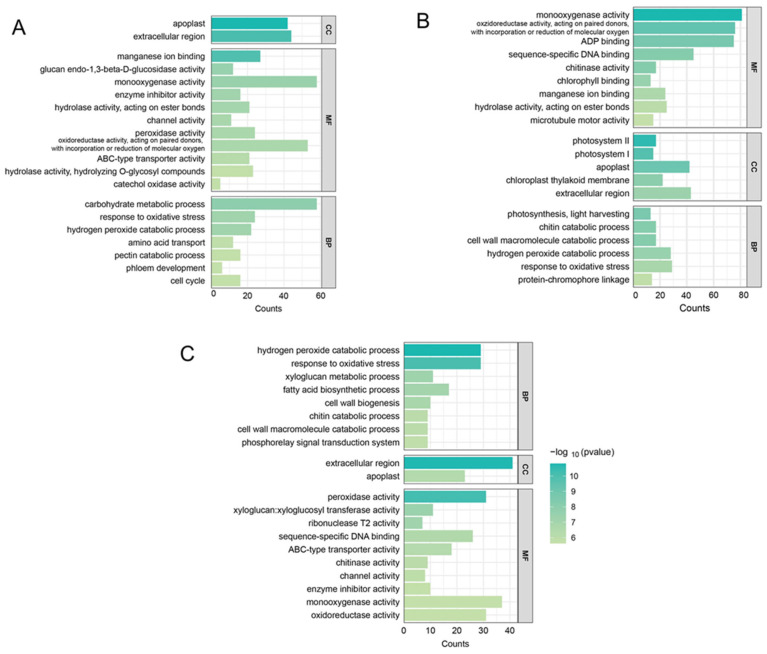
Bar charts of DEGs in GO enrichment analysis: (**A**) ZM1 vs. ZM2; (**B**) ZM1 vs. ZM3; (**C**) ZM2 vs. ZM3. In the figure, the horizontal axis shows the number of DEGs, and the vertical axis shows the Gene Ontology terms. ZM1, ZM2, and ZM3 correspond to callus induction, callus formation, and AR emergence stages, respectively.

To elucidate the major biological processes involved in AR formation, GO and KEGG pathway enrichment analyses were conducted on the DEGs identified across the three key developmental stages: 1924 DEGs from ZM1 vs. ZM2, 2473 DEGs from ZM1 vs. ZM3, and 1395 DEGs from ZM2 vs. ZM3 (Tables S1 and S2). For visualization, the top 20 most significantly enriched GO terms from the BP, CC, and MF categories were selected based on adjusted *p*-values (Figure 6). In the CC category, DEGs were predominantly enriched in components associated with extracellular processes across all three comparisons, suggesting key roles in signal transduction, ROS regulation, and cell wall remodeling during AR development. In the MF category, monooxygenase activity emerged as the most significantly enriched term. This highlights the role of DEGs in modulating hormone metabolism and maintaining ROS homeostasis, both critical for root initiation and development. In the BP category, distinct stage-specific enrichment patterns were observed: In ZM1 vs. ZM2, DEGs were significantly enriched in the carbohydrate metabolic process, indicating active energy metabolism to support rapid cell division and callus formation. In ZM2 vs. ZM3, DEGs were mainly enriched in processes such as the hydrogen peroxide catabolic process and response to oxidative stress, reflecting cellular efforts to degrade excess H_2_O_2_, thereby maintaining redox balance and establishing a favorable intracellular environment for root primordium development.

**Figure 7:**
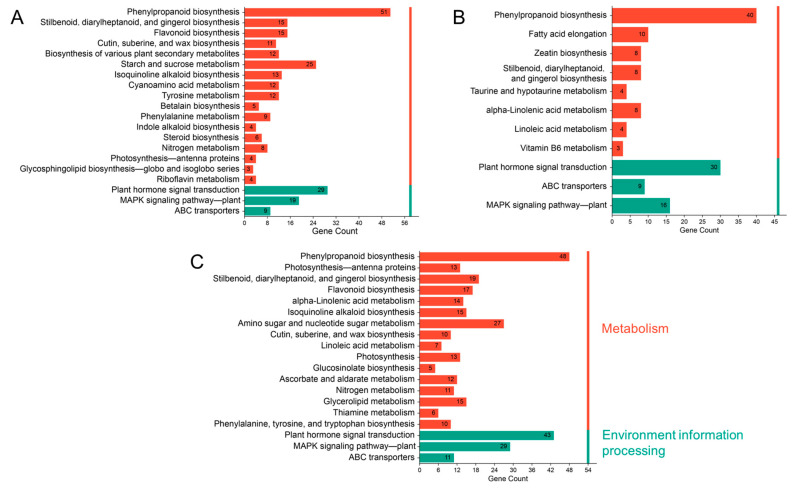
Bar charts of DEGs in KEGG enrichment analysis: (**A**) ZM1 vs. ZM2; (**B**) ZM1 vs. ZM3; (**C**) ZM2 vs. ZM3. In the figure, the horizontal axis shows the number of DEGs, the vertical axis shows the KEGG pathways, and the bar colors represent different categories of KEGG pathways. ZM1, ZM2, and ZM3 correspond to callus induction, callus formation, and AR emergence stages, respectively.

To further elucidate the regulatory transitions from the AR induction phase to root emergence, KEGG pathway enrichment analysis was performed on the DEGs from each pairwise comparison (Figure 7). A total of 45, 22, and 12 significantly enriched pathways were identified in the ZM1 vs. ZM2, ZM1 vs. ZM3, and ZM2 vs. ZM3 comparison groups, respectively. The top 20 significantly enriched pathways were selected for visualization. The results showed that most enriched pathways across all three comparison groups fell under the categories of metabolism and environmental information processing, particularly signal transduction. Among the metabolism-related pathways, phenylpropanoid biosynthesis was consistently enriched in all comparisons. In contrast, starch and sucrose metabolism was specifically enriched in the ZM1 vs. ZM2 group. Additionally, amino sugar and nucleotide sugar metabolism were notably enriched in the ZM1 vs. ZM3 comparison. Regarding signal transduction, two pathways—plant hormone signal transduction and the MAPK signaling pathway—were significantly enriched across all three stages, emphasizing their sustained involvement in coordinating hormone responses and cellular signaling during callus formation, root primordium induction, and AR emergence. These findings highlight the critical roles of both metabolic and signaling pathways in orchestrating the complex process of AR formation in *C. camphora*.

### 2.7. Analysis of DEGs Related to Nutrients and Oxidoreductases

**Figure 8:**
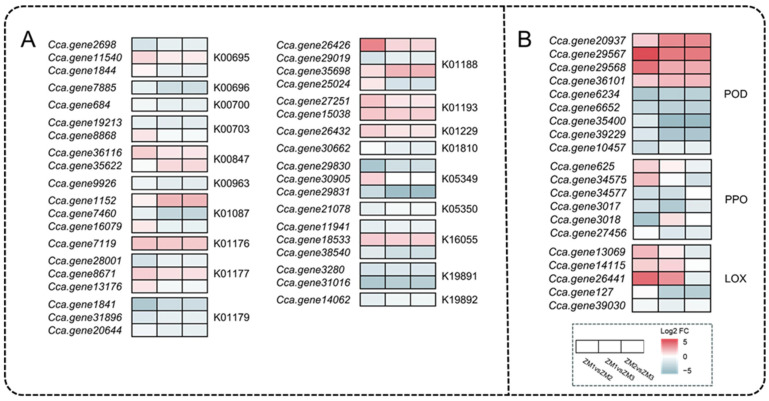
Heatmap analysis of DEGs related to related to starch and sucrose metabolism and redox-related enzymes. (**A**) DEGs related to starch and sucrose metabolism. (**B**) DEGs encoding redox-related enzymes. Colored boxes indicate the log_2_ fold changes in three comparison groups: ZM1 vs. ZM2, ZM1 vs. ZM3, and ZM2 vs. ZM3. Red, blue, and white boxes represent significantly upregulated, significantly downregulated, and non-significantly changed genes, respectively. The intensity of the color reflects the magnitude of the log_2_ fold change. ZM1, ZM2, and ZM3 correspond to callus induction, callus formation, and AR emergence stages, respectively.

Sugars serve not only as fundamental building blocks for plant life processes but also as the primary source of energy fueling plant growth and development. In the starch and sucrose metabolism pathway, 38 DEGs were involved in AR formation, primarily participating in sucrose metabolism, starch and polysaccharide metabolism, and trehalose metabolism (Figure 8A). In sucrose metabolism, two genes encoding beta-fructofuranosidase (INV, K01193) were significantly upregulated in the ZM1 vs. ZM2 and ZM1 vs. ZM3 comparison groups (Figure 8A and Figure S4). In addition, one gene encoding fructokinase (scrK, K00847) was significantly upregulated in the ZM1 vs. ZM2 and ZM2 vs. ZM3 comparison groups (Figure 8 and Figure S4).

In the phenylpropanoid biosynthesis pathway, the genes associated with POD activity exhibited divergent expression patterns (Figure 8B). Notably, five DEGs related to POD activity were significantly downregulated across all three comparisons, which is consistent with the observed decrease in POD activity. In addition, the gene (*Cca.gene3017*) encoding PPO was significantly downregulated across all three comparisons, in accordance with the overall downward trend of PPO activity (Figure 8B). As a key enzyme in lipid peroxidation, lipoxygenase (LOX) contributes to MDA production by catalyzing the oxidation of polyunsaturated fatty acids. Three genes encoding *LOX* were significantly upregulated in the ZM1 vs. ZM2 comparison, accompanied by an increase in MDA content (Figure 8B).

### 2.8. Analysis of DEGs in Key Signaling Pathways

**Figure 9:**
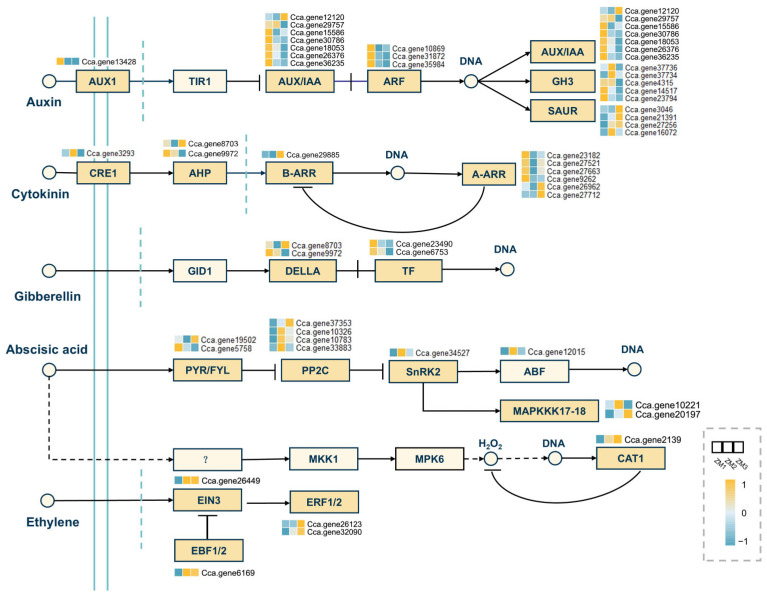
Expression analysis of DEGs in auxin, cytokinin, GA, ABA, and ET signal transduction pathways. The colored boxes represent the expression levels of DEGs; each box represents one time point. Yellow indicates high expression, and blue indicates low expression. The color gradient from blue to yellow indicates expression levels ranging from −1 to 1. ZM1, ZM2, and ZM3 correspond to callus induction, callus formation, and AR emergence stages, respectively.

KEGG enrichment analysis revealed that plant hormone signal transduction and MAPK signaling pathways were the most significantly enriched categories under environmental information processing across all comparison groups, underscoring their central regulatory roles during AR development. Therefore, we further analyzed gene expression profiles and regulatory functions associated with the five hormone signaling pathways, including auxin, cytokinin, ABA, GA and ET, within these two signaling pathways (Figure 9). A total of 67 DEGs were mapped to the plant hormone signal transduction pathway, including 20 genes specifically related to auxin signaling, highlighting auxin as the primary hormonal regulator in AR formation. Within the auxin pathway, most genes encoding *ARFs* and *Aux/IAA* family proteins were significantly downregulated across the three stages. In contrast, although several *Gretchen Hagen 3* (*GH3*) genes were upregulated during the callus formation stage, the majority showed an overall downregulation pattern during the AR emergence phase. Additionally, most *Small Auxin-Up RNA* (*SAUR*) genes exhibited upregulation throughout AR formation.

In the cytokinin signaling pathway, genes encoding *Type-A Arabidopsis Response Regulators* (*A-ARRs*) were significantly downregulated during both callus formation and AR emergence, suggesting a suppression of cytokinin signaling at these stages. Meanwhile, one *Type-B ARR* (*B-ARR*) gene showed upregulation. In the GA signaling pathway, two *DELLA* genes, known as negative regulators of GA signaling, were downregulated during callus formation, mirroring expression trends observed in several *transcription factor* (*TF*) genes. The MAPK signaling pathway, which integrates multiple hormonal cues such as ethylene (ET) and ABA, was also prominently enriched. A key gene involved in the ET signaling pathway, *ETHYLENE RESPONSE FACTOR 1* (*ERF1*), was significantly upregulated during callus formation. Conversely, several genes encoding *Protein Phosphatase 2C* (*PP2C*), negative regulators of ABA signaling, were downregulated during AR emergence. This likely resulted in the activation of *SNF1-related protein kinase 2* (*SnRK2*), thereby attenuating ABA signaling and facilitating root development (Figure 9).

**Figure 10:**
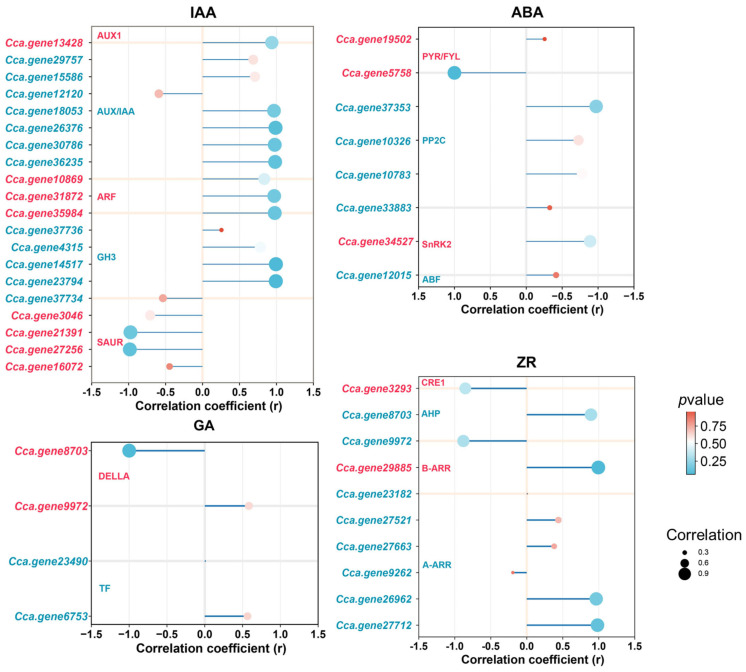
Correlation between the expression of auxin, cytokinin, gibberellin, and abscisic acid signaling pathway genes and the contents of endogenous hormones (IAA, ZR, GA, and ABA). The size of each dot represents the strength of the correlation between gene expression and endogenous hormone contents; larger dots indicate stronger correlations. The color of the dots reflects the *p*-value, with redder colors indicating smaller *p*-values.

Pearson correlation analysis revealed distinct associations between the levels of IAA, ABA, ZR, and GA and the expression of genes involved in the plant hormone signal transduction pathway during AR formation (Figure 10). Although IAA content consistently declined throughout the process, the expression levels of genes encoding *AUX/IAA*, *ARF*, and *GH3* in the auxin signaling pathway showed a significant positive correlation with IAA concentration. In contrast, *SAUR* gene expression exhibited a negative correlation with IAA levels. In the ABA signaling pathway, genes encoding *PP2C*, *SnRK2*, and *ABF* were positively correlated with endogenous ABA content, whereas *PYR/PYL* gene expression displayed a negative correlation. Within the CK signaling pathway, *CRE1* and *B-type ARR* genes were negatively correlated with ZR levels. Additionally, in the GA signaling pathway, the expression of *TF* genes demonstrated a positive correlation with GA levels.

### 2.9. Identification of Key Genes Associated with AR Formation

**Figure 11:**
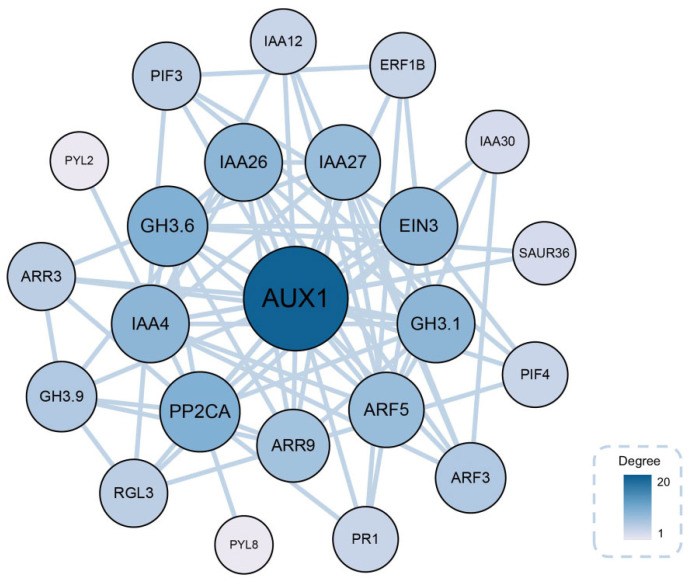
The diagram of protein network interactions. The size and color of each node represent the degree of the node, with larger and darker-colored nodes representing higher degrees of connectivity.

To further elucidate the role of endogenous hormones in AR formation in tissue-cultured *C. camphora* plantlets, DEGs involved in plant hormone signal transduction were subjected to protein–protein interaction (PPI) network analysis to identify key regulatory nodes. Proteins exhibiting high connectivity within the PPI network were considered potential hub proteins essential for AR development (Table S3). Corresponding genes encoding these hub proteins were further recognized as key regulatory elements in the molecular mechanisms underlying AR formation. Based on this analysis, ten candidate genes were identified as central regulators of rooting. These included genes encoding *CcAUX1*, *CcPP2CA*, *CcGH3.6*, *CcEIN3*, *CcIAA26*, *CcIAA4*, *CcGH3.1*, *CcARF5*, *CcIAA27*, and *CcARR9* (Figure 11).

### 2.10. Validation of Gene Expression

**Figure 12:**
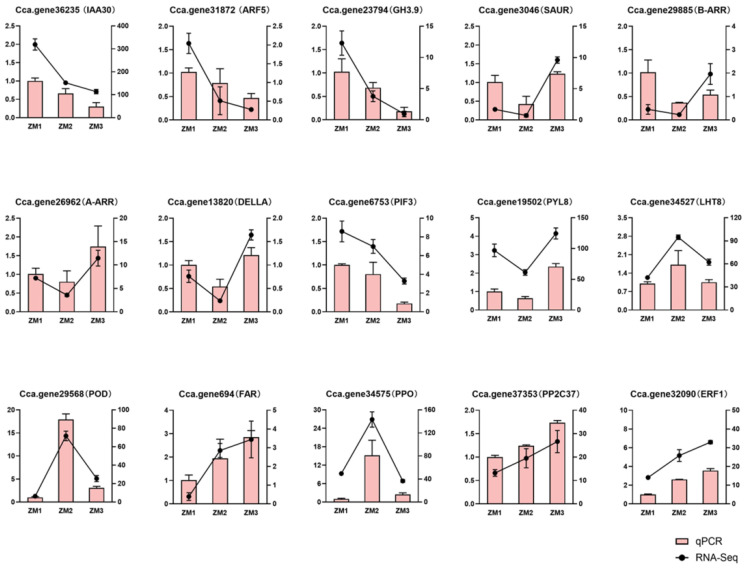
The qRT-PCR validation of genes selected randomly in RNA-seq results. Each cell includes the values at different time points. Pink bars show the relative expression levels by qRT-PCR. Blank lines represent the FPKM of RNA-seq. ZM1, ZM2, and ZM3 correspond to callus induction, callus formation, and AR emergence stages, respectively.

To verify the accuracy of the transcriptomic data, fifteen rooting-related genes were selected from the DEGs for validation using qRT-PCR. The expression patterns obtained from qRT-PCR analysis were largely consistent with those from RNA-seq (Figure 12).

## 3. Discussion

### 3.1. The Process of AR Formation in C. camphora

In most spermatophytes, ARs can form either constitutively or in response to external stimuli such as mechanical injury, flooding, biotic stress, or hormonal signals, particularly under tissue culture conditions [37]. ARs are typically categorized into latent or induced types, depending on their developmental origin. Anatomical observations in this study revealed the absence of latent root primordia at the base of *C. camphora* stem segments, indicating that AR formation follows an induced pathway. Based on the site of root primordium initiation, AR formation is further classified into three types: cortex-rooting, callus-rooting, and mixed-rooting [38]. Our findings showed that root primordia in *C. camphora* originated from callus tissue, suggesting that this species undergoes callus-type rooting. This pattern aligns with previous reports on *C. parthenoxylon* cuttings [39]. However, ARs formed through callus induction typically exhibit lower mechanical strength, rendering them more susceptible to breakage during transplantation and potentially reducing post-transplant survival rates.

### 3.2. Regulation Mechanism of Nutrients and Oxidoreductases During AR Formation

During AR formation, cell division and cell enlargement require a high input of energy and carbon skeletons. Sucrose concentration in the culture medium directly influences endogenous carbohydrate levels and increases subsequent root formation [40,41]. As the major carbon source, sucrose is hydrolyzed into hexoses—mainly glucose and fructose—which subsequently enter the glycolytic pathway to produce ATP [42]. During AR formation in *C. camphora* tissue culture, genes encoding INV were significantly upregulated, catalyzing the hydrolysis of sucrose into glucose and fructose—a pivotal step in sucrose catabolism [43]. The liberated fructose was subsequently phosphorylated by scrK to generate fructose-6-phosphate, a key intermediate of the glycolytic pathway [44]. The marked upregulation of *scrK* observed in this study suggests an enhanced flux through glycolysis, reflecting the increased demand for energy and carbon skeletons during root induction and development. The progressive decline in soluble protein content observed in this study may reflect the high metabolic demand for protein synthesis during cell differentiation and tissue remodeling, a phenomenon similarly reported in *C. parthenoxylon* [39].

AR formation was also accompanied by marked fluctuations in redox status, as indicated by changes in MDA content and antioxidant enzyme activity [45]. As a key enzyme in lipid peroxidation, LOX is closely linked to MDA accumulation [46]. In this study, *LOX*-encoding genes were markedly upregulated at the early stage of AR formation, corresponding to a notable increase in MDA levels, implying a moderate rise in ROS, which are known to function as signaling molecules during organogenesis. This increase coincided with a transient decline in SOD and POD activities. Genes encoding *CcPOD16*, *CcPOD42*, and *CcPOD68* were significantly downregulated, consistent with the observed decrease in POD activity. These changes in enzyme activities suggest a controlled relaxation of the antioxidant defense system to permit ROS-mediated signaling during callus and root primordium formation [47]. Species within the genus *Cinnamomum* are known to secrete phenolic compounds from their roots, such as phenols [48]. PPOs catalyze the oxidation of phenols into toxic o-quinones, which can impair tissue viability [49]. To mitigate this effect, AC was incorporated into the culture medium to adsorb phenolic compounds, thereby lowering PPO substrate availability and reducing browning reactions [50].

### 3.3. Regulation Mechanism of Auxin

AR formation is predominantly regulated by the balance between endogenous and exogenous phytohormones during tissue culture [37]. In vitro, AR induction is particularly difficult in the absence of exogenous hormone supplementation. To address this challenge, stem segment explants are typically cultured on media supplemented with various auxins or their synthetic analogs, such as IAA, IBA, and NAA [13]. Among these, NAA is widely utilized due to its efficacy in promoting AR development; for example, exogenous application of NAA has been shown to significantly enhance AR formation in *Robinia pseudoacacia* [51]. Similarly, in the present study, ARs were successfully induced in *C. camphora* plantlets cultured on a rooting medium containing NAA. Following NAA application, a continuous decline in endogenous IAA levels was observed, suggesting that NAA may inhibit IAA biosynthesis via negative feedback regulation. In *Arabidopsis*, the expression levels of *Tryptophan Aminotransferase Related* (*TAR*) and *YUCCA(YUC)* genes are downregulated in response to exogenous NAA application, which is consistent with the downregulation of *CcYUC10* observed throughout the AR formation process in this study [52].

Auxin functions as the primary regulator of AR formation by acting on rooting-competent tissues, determining cell fate, and activating downstream signaling networks [53]. Given the central role of auxin, we further explored the auxin signaling pathway in this study. Additionally, KEGG pathway analysis revealed that the auxin signaling pathway harbored the highest number of DEGs, underscoring its critical involvement in AR regulation. Correlation analysis showed that the expression of *CcAUX/IAA*, *CcARF*, and *CcGH3* genes was positively correlated with IAA levels, whereas members of the *CcSAUR* gene family were negatively correlated. The initiation and modulation of auxin signaling are orchestrated by two key transcription factor gene families: *AUX/IAA* and *ARF*. In *Populus*, auxin-induced repression of AR development by *IAA4* overexpression can be partially alleviated through exogenous auxin application [54]. In our study, NAA treatment enhanced auxin signaling by promoting the ubiquitin-mediated degradation of *AUX/IAA* repressors via the 26S proteasome, thereby releasing ARF transcriptional activity. Six auxin-responsive *AUX/IAA* genes (*CcIAA4*, *CcIAA12*, *CcIAA25*, *CcIAA26*, *CcIAA27*, and *CcIAA30*) were differentially expressed, exhibiting a consistent downward trend during AR formation. ARF proteins are divided into two functional classes: activators containing Q-rich domains that promote the expression of auxin-responsive genes, and repressors that inhibit their transcription [55]. In this study, the expression of *CcARF3* and *CcARF5* was significantly downregulated during AR formation. This trend is consistent with prior findings in rice, where *OsARF5* showed a moderate decrease following auxin treatment during root development [56]. Notably, *CcARF3* has been characterized as a transcriptional repressor, and its expression profile aligns with previously reported data [57]. In addition to *AUX/IAA* and *ARF*, the *GH3* and *SAUR* gene families are crucial components of the auxin signaling cascade. GH3 enzymes conjugate excess IAA to amino acids, acting as negative regulators to maintain auxin homeostasis, whereas SAUR genes are typically upregulated in response to auxin and promote cell elongation during AR development [58,59]. Specifically, *CcGH3.9* likely acts as a buffering factor within the auxin pathway, potentially inhibiting primary root elongation, with its expression pattern consistent with previous studies [60]. Furthermore, *CcSAUR36* has been shown to be rapidly induced by NAA treatment, supporting its potential role in NAA-mediated AR formation [61].

### 3.4. Regulation Mechanism of Endogenous ZR

Auxin interacts intricately with other phytohormones through complex regulatory networks, exhibiting dynamic crosstalk and antagonistic relationships [13]. Among these, auxin and cytokinin often function antagonistically in various physiological processes, including AR formation [62]. Exogenous auxin treatment typically initiates organogenic responses, accompanied by the production of endogenous cytokinins and localized activation of cytokinin signaling pathways [63]. ZR, a key cytokinin species, has been identified as a major inhibitor of AR formation in cucumber [30]. In this study, ZR content gradually declined during callus formation and sharply increased during AR emergence. These results suggest that exogenous auxin may suppress ZR accumulation during the early callus stage, while ZR may later promote cell division and contribute to root primordium initiation during AR emergence. Following cytokinin perception, cytokinin receptors—*ARABIDOPSIS HISTIDINE KINASEs (AHKs)*—autophosphorylate and transfer phosphate groups to *ARABIDOPSIS HISTIDINE PHOSPHOTRANSFER PROTEINs (AHPs)* [64]. These *AHPs* then translocate to the nucleus and phosphorylate *ARABIDOPSIS RESPONSE REGULATORs (ARRs)*, including both type-A and type-B ARR proteins [65]. In our transcriptome data, the gene encoding the type-A ARR *CcARR9* showed an upregulated trend, suggesting that auxin may inhibit cytokinin signaling by modulating *CcA-ARR* expression and downregulating *CcAHP1*. This suppression of cytokinin signaling could alleviate its inhibitory effect on AR formation.

### 3.5. Regulation Mechanism of Endogenous ABA and ET

As a stress-responsive inhibitory hormone, elevated levels of ABA can disrupt the biosynthesis and transport of IAA, thereby impairing normal plant growth and AR formation [66]. The ABA signaling pathway is mediated by a core module consisting of pyrabactin resistance/pyrabactin resistance-like/regulatory components of ABA receptors (PYR/PYL/RCAR), PP2Cs, and SnRK2s [67]. Activation of this module results in the phosphorylation of downstream targets, including ABA-responsive element binding factors (AREBs/ABFs), which subsequently regulate the expression of ABA-responsive genes [68]. In this study, endogenous ABA levels continuously declined throughout AR formation, a trend consistent with previous reports. Expression of *PYR/PYL* genes was downregulated during the callus formation stage, in parallel with decreasing ABA content, indicating attenuation of ABA signaling in the early phase of root induction. Notably, *CcPP2C37* was significantly upregulated during AR development, suggesting a negative feedback regulation mechanism within the ABA signaling pathway [69]. Meanwhile, *CcSnRK2* expression exhibited an upward trend. As a key regulator of root development, *SnRK2* overexpression has been shown to promote primary root elongation and improve abiotic stress tolerance in *Triticum aestivum* transgenic lines [70]. However, the precise roles of ABA-independent functions of *SnRK2s* remain largely uncharacterized. In this study, among the PP2C family members identified, three *CcPP2C51* genes displayed a dynamic expression pattern: they were upregulated during the callus induction and formation stages, followed by a sharp decrease during AR emergence. This trend mirrored that of the *ABF* gene. Additionally, ABA signaling is known to interact with MAPK cascades, which convert receptor-mediated cues into a range of cellular responses [71]. The MAPK cascade typically includes a three-tiered module: MAPKKKs, MKKs, and MPKs. Although MAPKKKs constitute the largest subgroup, only a few have been implicated in ABA-related signaling. For instance, *CcMAPKKK18* has been reported to mediate ABA responses [72], and ABF transcription factors are known to regulate *MAPKKK18* expression, thereby linking ABA signaling to cellular senescence [73].

In tissue culture conditions, the use of an agar-based medium results in reduced aeration, which can stimulate ET biosynthesis and accumulation in plant tissues [74]. During the early stage of AR induction, two genes (*Cca.gene15822* and *Cca.gene15824*) encoding *1-aminocyclopropane-1-carboxylic acid synthase* (*ACS*), a key rate-limiting enzyme in the ET biosynthetic pathway, were significantly upregulated, suggesting a potential increase in ET production [75]. In the ET signaling pathway, *ETHYLENE INSENSITIVE 3* (*EIN3*) functions as a central transcription factor and regulator of ET responses [76]. It promotes the expression of downstream genes, including *ETHYLENE RESPONSE FACTOR* (*ERF*) family members [77]. In this study, both *EIN3* and *ERF* genes were upregulated during AR formation, suggesting that ET likely facilitates callus development and AR emergence in *C. camphora* under tissue culture conditions.

These findings provide experimental evidence for optimizing the regeneration system of *C. camphora* and enhance our theoretical understanding of AR development. The regulatory network was primarily analyzed at the levels of anatomical observation, physiological traits, endogenous hormones, and transcriptomic profiling. To gain a more comprehensive understanding of the molecular mechanisms underlying AR formation, future studies should integrate proteomic and metabolomic approaches to identify key regulators and construct a systematic regulatory network. Such multi-omics integration will contribute to a deeper and more holistic understanding of *C. camphora* tissue culture and AR development.

## 4. Materials and Methods

### 4.1. Plant Materials

Tissue culture-derived seedlings of *C. camphora* were obtained from the Modern Plant Tissue Culture and Breeding Center at the Jiangxi Academy of Forestry (115°48′47″ E, 28°44′41″ N) and used as experimental materials. Explants grown under optimal conditions were subcultured four times on a proliferation medium (MS supplemented with 2.0 mg/L 6-BA, 0.1 mg/L zeatin [ZT], and 0.5 mg/L indole-3-butyric acid [IBA]) at 35-day intervals to induce adventitious shoot clusters. Subsequently, lignified shoots (3–4 cm in length) were excised and transferred to a rooting medium (1/2 MS supplemented with 1.0 mg/L NAA and 0.5 g/L AC) for a 30-day induction period. Sampling time points were determined based on visible morphological changes. At 3, 7, and 10 days during AR formation, the stem segments (~1 cm) from the basal region of the shoots, where ARs primarily formed, were collected to represent three developmental stages: callus induction (ZM1), callus formation (ZM2), and AR emergence (ZM3). Basal segments were used for anatomical observations, physiological measurements, and transcriptomic analysis. Each experimental group included three biological replicates. Samples of three stages were collected at each stage, immediately frozen in liquid nitrogen, and stored at −80 °C until subsequent unified analysis.

### 4.2. Morphological and Anatomical Observation

Paraffin sectioning was performed to examine anatomical changes during root formation. Approximately 5 mm of tissue was excised from the basal region of explants and fixed in FAA solution for 48 h at 4 °C to preserve cellular structure. Samples were then dehydrated through a graded ethanol series, embedded in paraffin, and stained with safranin and fast green. The stained sections were mounted in neutral resin and observed under a DFC500 optical microscope (Leica Microsystems, Wetzlar, Germany). Representative anatomical structures were imaged, and key features were documented.

### 4.3. Physiological Index Measurement

Soluble sugar content was determined using the anthrone colorimetric method, and soluble protein content was measured by the Coomassie Brilliant Blue assay. Endogenous hormone levels—including IAA, GA, ABA, and ZR—in the phloem of the basal stem segments were quantified using high-performance liquid chromatography–mass spectrometry (HPLC-MS). MDA content was analyzed using the thiobarbituric acid (TBA) method. The enzymatic activities of SOD, PPO, and POD were assessed using standard colorimetric assays. Each sample was analyzed with six biological replicates.

### 4.4. RNA-Seq and Data Analysis

Total RNA was extracted from approximately 100 mg of ground plant tissue using the Omega Plant RNA Kit (Omega Bio-Tek, Norcross, GA, USA), following the manufacturer’s protocol. Briefly, tissue was homogenized in liquid nitrogen, lysed in RB buffer containing β-mercaptoethanol, passed through a gDNA removal column, and purified using spin columns with ethanol-based precipitation. RNA integrity was verified by 1% agarose gel electrophoresis, and purity and concentration were measured using a NanoDrop spectrophotometer (Thermo Fisher Scientific, Waltham, MA, USA). High-quality RNA samples were used for cDNA library construction, which was performed by Wuhan Benagen Technology Co., Ltd. (Wuhan, China). After quality control, libraries were sequenced using the DNBSEQ-T7 high-throughput sequencing platform. Three biological replicates were included for each group. Raw sequencing data were subjected to quality control using Fastp [78]. Reads containing more than 5% unknown nucleotides (N) or with over 50% low-quality bases (Q ≤ 20) were removed to obtain high-quality clean reads. Clean reads were aligned to the *C. camphora* reference genome using STAR, and sequencing quality was further assessed through randomness and saturation analyses. Transcript quantification was conducted using RSEM, generating both read counts and FPKM values. Paired-end reads from the same fragment were treated as a single sequencing unit.

### 4.5. Identification and Analysis of DEGs

DEGs among different rooting stages were identified using DESeq2, with thresholds of |log2FC| > 1 and adjusted *p*-value (adj) < 0.05. To examine dynamic expression patterns across time points, fuzzy clustering analysis was conducted using the Mfuzz package (Short Time-series Expression Miner, version 2.68.0). Functional enrichment analysis, including GO and KEGG pathway analyses, was then performed for each comparison. The Pearson correlation coefficient was used to evaluate the association between expression changes of DEGs in the auxin, cytokinin, GA, and ABA signaling pathways and the corresponding endogenous hormone levels. Then, the STRING database was used for PPI analysis of DEGs in the auxin, cytokinin, GA, ABA, and ET signaling pathways. The cytohubba plugin in Cytoscape (version 3.10.2) was employed to calculate the degree values of the nodes in the PPI network and visualize the network to identify key nodes.

### 4.6. Quantitative Real-Time PCR (qRT-PCR) Validation

Selected DEGs related to AR formation were validated by qRT-PCR. Gene-specific primers were designed using Primer Premier 5 (Table S4) and synthesized by Ruibiotech (Beijing, China). Actin was used as the internal reference gene. Relative gene expression was calculated using the 2^−ΔΔCT^ method. All reactions were conducted in triplicate with three independent biological replicates.

### 4.7. Statistical Analysis

Statistical significance was assessed utilizing one-way analysis of variance (ANOVA), followed by the least significant difference (LSD) post hoc test, with a threshold of *p* < 0.05. All statistical analyses were implemented utilizing SPSS (version 26), and graphical representations were created using GraphPad Prism (version 9.0).

## 5. Conclusions

In this study, detailed morphological and anatomical analyses revealed that ARs in *C. camphora* primarily originate from callus tissue under tissue culture conditions. Significant changes in nutrient levels, endogenous hormone concentrations, and antioxidant enzyme activities were observed during AR formation. A decline in SOD and POD activities, coupled with increased MDA levels from ZM1 to ZM2, indicated a moderate accumulation of ROS. This oxidative stress appears to promote callus development and root primordium differentiation. Transcriptome analysis using Mfuzz clustering grouped DEGs into four temporal expression patterns. KEGG pathway enrichment further revealed their stage-specific involvement in signal transduction, energy metabolism, cytoskeletal organization, and antioxidant responses, highlighting the dynamic regulation of gene expression during AR formation. A total of 67 DEGs were enriched in the plant hormone signal transduction pathway, including 20 genes specifically related to auxin signaling, confirming auxin as the central hormonal regulator in this process. Correlation analysis showed that auxin-repressive genes such as *AUX/IAA* were positively associated with IAA levels, suggesting that exogenous NAA, a synthetic auxin analog, may function as both a substitute for and a modulator of endogenous auxin signaling. Furthermore, significant transcriptional changes were also observed in genes involved in cytokinin, ABA, and ET signaling pathways, indicating a coordinated hormonal network regulating AR formation. Overall, this study provides a comprehensive transcriptomic framework for understanding the molecular mechanisms underlying AR formation in *C. camphora*. The findings contribute valuable insights into key regulatory genes and offer a theoretical foundation for improving rooting efficiency and optimizing in vitro propagation systems.

## Data Availability

The raw sequence data reported in this paper have been deposited in the Genome Sequence Archive (*Genomics, Proteomics & Bioinformatics* 2021) in the National Genomics Data Center (*Nucleic Acids Res* 2024), China National Center for Bioinformation/Beijing Institute of Genomics, Chinese Academy of Sciences (CRA019667), and are publicly accessible at https://bigd.big.ac.cn/gsa/browse/CRA019667, accessed on 17 October 2024.

## Supplementary Materials

The following supporting information can be downloaded at https://www.mdpi.com/article/10.3390/ijms26157264/s1.
