# Supplementary material for: Physiological and Transcriptomic Analyses Reveal Regulatory Mechanisms of Adventitious Root Formation in In Vitro Culture of Cinnamomum camphora
Source: Int J Mol Sci. 2025 Jul 27;26(15):7264. doi: 10.3390/ijms26157264 (PMC12347477; doi:10.3390/ijms26157264)
Supplement: Supplementary file 1 [file ijms-26-07264-s001.zip › ijms-3726620-supplementary.pdf]

Supplementary Materials

S1.1. Supplementary Figures

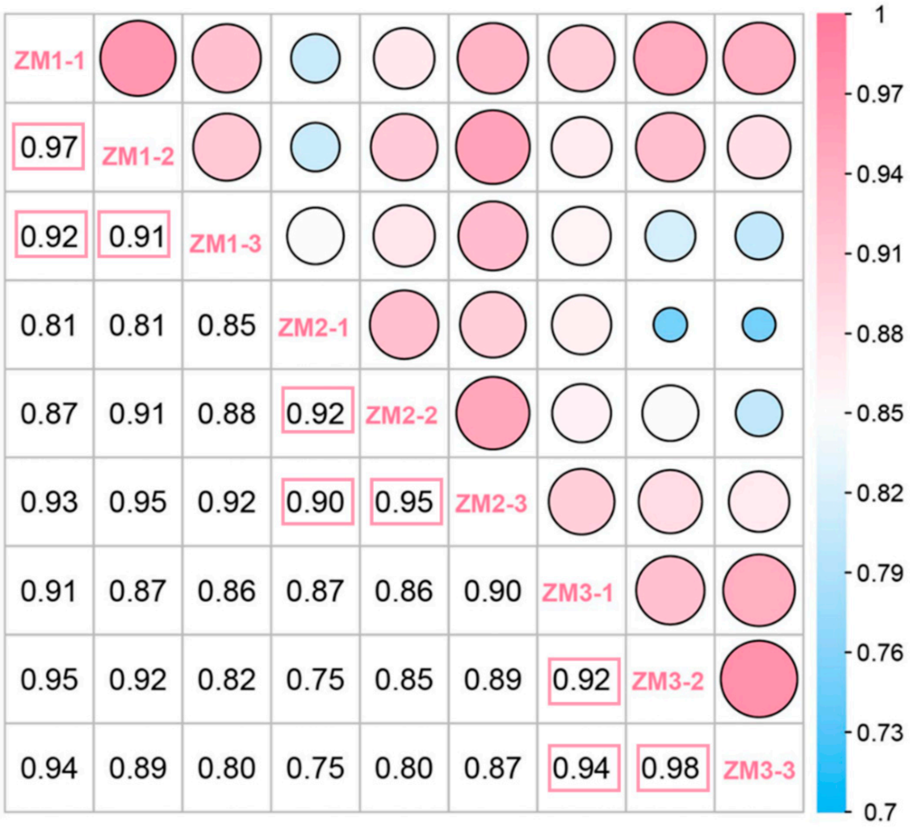

Figure S1. The correlation of samples.

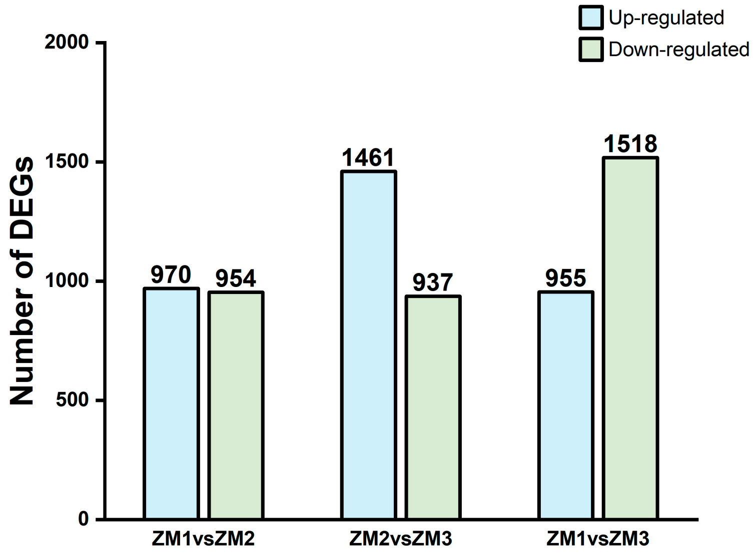

Figure S2. The numbers of DEGs in the three comparison groups. ZM1, ZM2, and ZM3 correspond to callus induction, callus formation, and AR emergence stages, respectively.

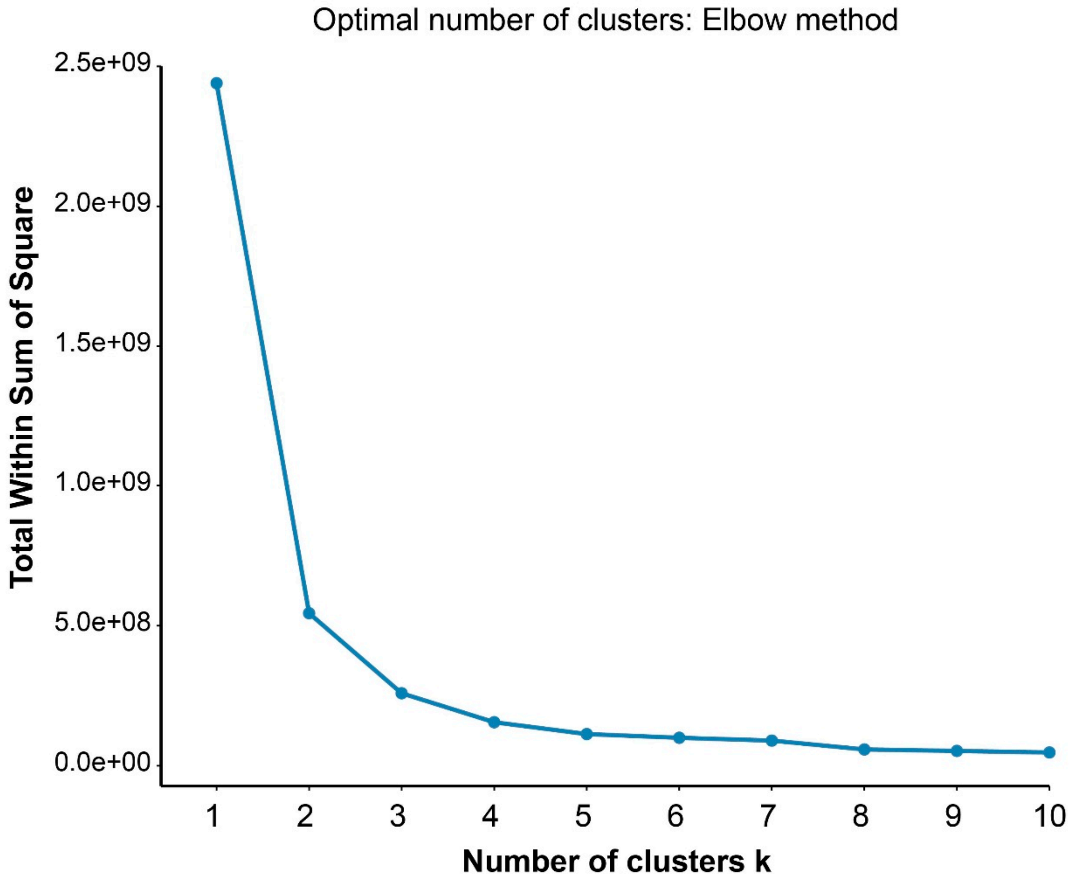

Figure S3. Elbow plot for determining the optimal number of clusters (k = 6).

A

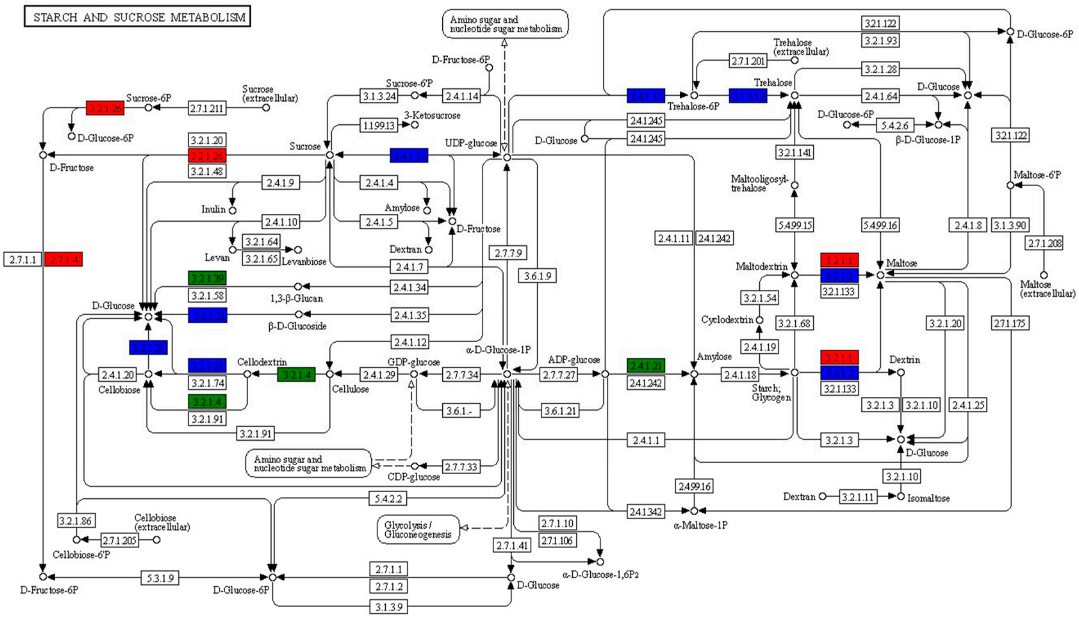
STARCH AND SUCROSE METABOLISM
Amino sugar and nucleotide sugar metabolism
D-Fructose-6P
Sucrose-6P
Sucrose (extracellular)
D-Glucose-6P
D-Fructose
Sucrose
3-Ketosucrose
UDP-glucose
D-Glucose
Trehalose (extracellular)
Trehalose
Trehalose-6P
D-Glucose-6P
β-D-Glucose-1P
Inulin
Amylose
D-Fructose
Levan
Levanbiose
Dextran
Maltooligosyl-trehalose
Maltose-6P
Maltodextrin
Maltose
Maltose (extracellular)
1,3-β-Glucan
β-D-Glucoside
Cyclodextrin
α-D-Glucose-1P
ADP-glucose
Amylose
Starch; Glycogen
Dextrin
Cellodextrin
GDP-glucose
Cellobiose
Cellulose
D-Glucose
Amino sugar and nucleotide sugar metabolism
CDP-glucose
Dextran
Isomaltose
Cellobiose (extracellular)
Cellobiose-6P
Glycolysis / Gluconeogenesis
α-Maltose-1P
α-D-Glucose-1,6P2
D-Fructose-6P
D-Glucose-6P
D-Glucose

B

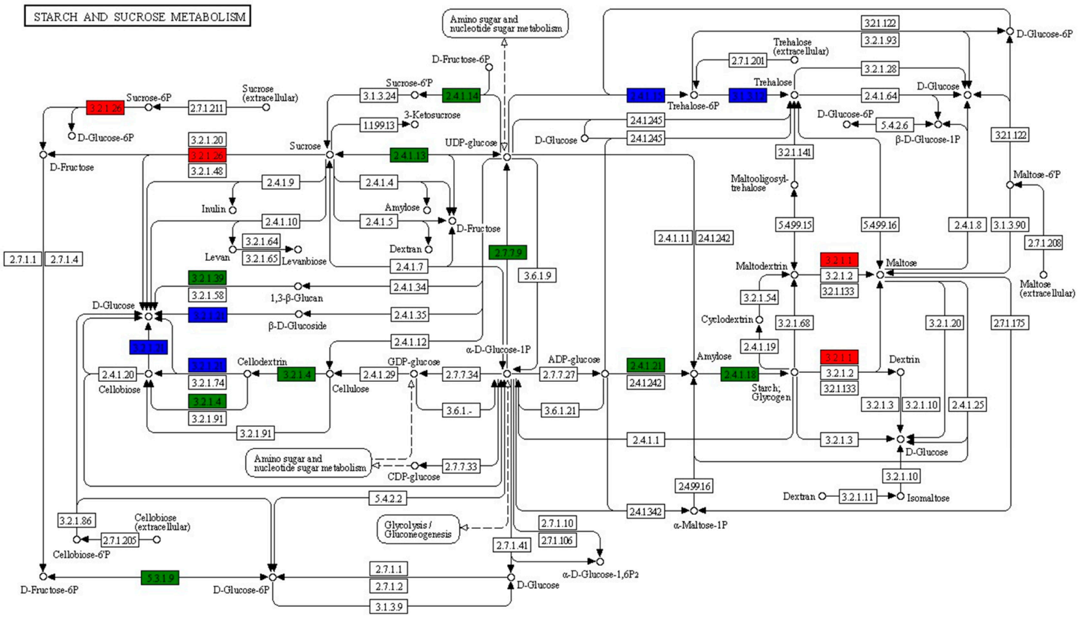
STARCH AND SUCROSE METABOLISM
Amino sugar and nucleotide sugar metabolism
D-Fructose-6P
Sucrose-6P
Sucrose (extracellular)
D-Glucose-6P
D-Fructose
Sucrose
3-Ketosucrose
UDP-glucose
D-Glucose
Trehalose (extracellular)
Trehalose
Trehalose-6P
D-Glucose-6P
β-D-Glucose-1P
Inulin
Amylose
D-Fructose
Levan
Levanbiose
Dextran
Maltooligosyl-trehalose
Maltose-6P
Maltodextrin
Maltose
Maltose (extracellular)
1,3-β-Glucan
β-D-Glucoside
Cyclodextrin
α-D-Glucose-1P
ADP-glucose
Amylose
Starch; Glycogen
Dextrin
Cellodextrin
GDP-glucose
Cellobiose
Cellulose
D-Glucose
Amino sugar and nucleotide sugar metabolism
CDP-glucose
Dextran
Isomaltose
Cellobiose (extracellular)
Cellobiose-6P
Glycolysis / Gluconeogenesis
α-Maltose-1P
α-D-Glucose-1,6P2
D-Fructose-6P
D-Glucose-6P
D-Glucose

C

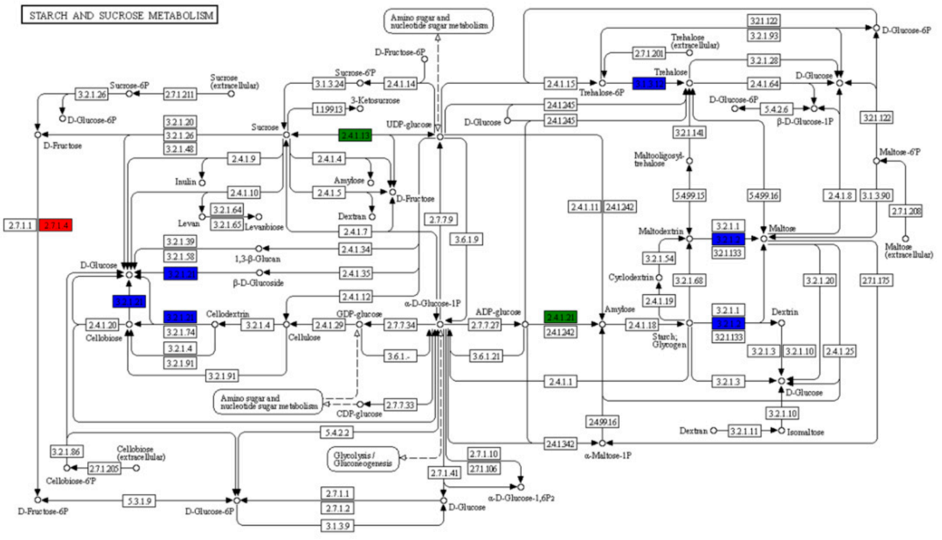

Figure S4. Anotation of DEGs of comparison groups in the starch and sucrose metabolism pathway. (A) ZM1 vs. ZM2. (B) ZM1 vs. ZM3 (C) ZM2 vs. ZM3. Greeen indicates that all the DEGs encoding this protein are downregualted, red indicates that all DEGs encoding the protein are upgualted, and blue indicates that DEGs encoding the protein are both upregulated and downregualed. ZM1, ZM2, and ZM3 correspond to callus induction, callus formation, and AR emergence stages, respectively.

S1.2. Supplementary Tables

Table S1. GO enrichment analyses of DEGs.

Group	ID	Description	GeneRatio	pvalue	Count
ZM1 vs. ZM2	GO:0048046	apoplast	42/1481	1.57E-13	42
	GO:0005576	extracellular region	44/1481	3.72E-12	44
	GO:0030145	manganese ion binding	27/1481	6.87E-12	27
	GO:0005975	carbohydrate metabolic process	58/1481	3.37E-08	58
	GO:0042973	glucan endo-1,3-beta-D-glucosidase activity	12/1481	1.64E-07	12
	GO:0004497	monooxygenase activity	58/1481	1.93E-07	58
	GO:0006979	response to oxidative stress	24/1481	6.39E-07	24
	GO:0004857	enzyme inhibitor activity	16/1481	7.11E-07	16
	GO:0042744	hydrogen peroxide catabolic process	22/1481	8.13E-07	22
	GO:0016788	hydrolase activity, acting on ester bonds	21/1481	2.00E-06	21
	GO:0015267	channel activity	11/1481	2.96E-06	11
	GO:0004601	peroxidase activity	24/1481	4.35E-06	24
	GO:0016705	oxidoreductase activity, acting on paired donors, with incorporation or reduction of molecular oxygen	53/1481	4.48E-06	53

GO:0140359	ABC-type transporter activity	21/1481	3.41E-05	21
GO:0006865	amino acid transport	12/1481	0.000211137	12
GO:0004553	hydrolase activity, hydrolyzing O-glycosyl compounds	23/1481	0.000401859	23
GO:0004097	catechol oxidase activity	5/1481	0.000442368	5
GO:0045490	pectin catabolic process	16/1481	0.000444506	16
GO:0010088	phloem development	6/1481	0.000475365	6
GO:0007049	cell cycle	16/1481	0.00049637	16
GO:0004089	carbonate dehydratase activity	7/1481	0.000688281	7
GO:0009664	plant-type cell wall organization	8/1481	0.000769221	8
GO:0015074	DNA integration	13/1481	0.001085393	13
GO:0005507	copper ion binding	13/1481	0.001219837	13
GO:0043565	sequence-specific DNA binding	26/1481	0.001240003	26
GO:0030599	pectinesterase activity	13/1481	0.002117282	13
GO:0042545	cell wall modification	13/1481	0.002117282	13
GO:0045330	aspartyl esterase activity	13/1481	0.002117282	13
GO:0016762	xyloglucan:xyloglucosyl transferase activity	7/1481	0.00308643	7
GO:0050136	NADH dehydrogenase (quinone) activity	4/1481	0.003105465	4
GO:0005886	plasma membrane	38/1481	0.004149509	38
GO:0016831	carboxy-lyase activity	5/1481	0.0057759	5
GO:0016760	cellulose synthase (UDP-forming) activity	10/1481	0.006004529	10
GO:0010181	FMN binding	6/1481	0.006876092	6
GO:0008061	chitin binding	5/1481	0.007209513	5
GO:0003777	microtubule motor activity	8/1481	0.007562343	8
GO:0008271	secondary active sulfate transmembrane transporter activity	4/1481	0.008143543	4
GO:0051301	cell division	16/1481	0.009226486	16
GO:0030244	cellulose biosynthetic process	10/1481	0.010022461	10
GO:0008289	lipid binding	14/1481	0.010234313	14
GO:0006284	base-excision repair	4/1481	0.010581178	4
GO:0006817	phosphate ion transport	4/1481	0.010581178	4
GO:0009734	auxin-activated signaling pathway	13/1481	0.011491263	13
GO:0008017	microtubule binding	14/1481	0.013561174	14
GO:0010411	xyloglucan metabolic process	6/1481	0.015065771	6
GO:0016614	oxidoreductase activity, acting on CH-OH group of donors	6/1481	0.015065771	6
GO:0042546	cell wall biogenesis	6/1481	0.015065771	6
GO:0031047	gene silencing by RNA	7/1481	0.017497009	7
GO:0007018	microtubule-based movement	9/1481	0.018957572	9
GO:0009873	ethylene-activated signaling pathway	8/1481	0.020580346	8
GO:0004190	aspartic-type endopeptidase activity	11/1481	0.021109156	11
GO:0008610	lipid biosynthetic process	5/1481	0.024562285	5
GO:0040029	regulation of gene expression, epigenetic	3/1481	0.025181416	3
GO:0022857	transmembrane transporter activity	22/1481	0.031490271	22
GO:0008308	voltage-gated anion channel activity	3/1481	0.032096217	3

	GO:0005874	microtubule	12/1481	0.033761501	12
	GO:0004650	polygalacturonase activity	9/1481	0.033799551	9
	GO:0004568	chitinase activity	6/1481	0.039153869	6
	GO:0006032	chitin catabolic process	6/1481	0.039153869	6
	GO:0016998	cell wall macromolecule catabolic process	6/1481	0.039153869	6
	GO:0035556	intracellular signal transduction	4/1481	0.040571485	4
	GO:0009523	photosystem II	5/1481	0.041317332	5
	GO:0009765	photosynthesis, light harvesting	4/1481	0.046842282	4
	GO:0016702	oxidoreductase activity, acting on single donors with incorporation of molecular oxygen, incorporation of two atoms of oxygen	4/1481	0.046842282	4
	GO:0045087	innate immune response	3/1481	0.048552533	3
ZM2 vs. ZM3	GO:0042744	hydrogen peroxide catabolic process	29/1062	3.60E-15	29
	GO:0005576	extracellular region	41/1062	4.79E-15	41
	GO:0004601	peroxidase activity	31/1062	6.55E-14	31
	GO:0006979	response to oxidative stress	29/1062	1.53E-13	29
	GO:0016762	xyloglucan:xyloglucosyl transferase activity	11/1062	7.70E-08	11
	GO:0010411	xyloglucan metabolic process	11/1062	1.09E-07	11
	GO:0033897	ribonuclease T2 activity	7/1062	1.84E-07	7
	GO:0006633	fatty acid biosynthetic process	17/1062	3.85E-07	17
	GO:0042546	cell wall biogenesis	10/1062	1.13E-06	10
	GO:0043565	sequence-specific DNA binding	26/1062	5.99E-06	26
	GO:0048046	apoplast	23/1062	8.78E-06	23
	GO:0140359	ABC-type transporter activity	18/1062	1.31E-05	18
	GO:0004568	chitinase activity	9/1062	6.36E-05	9
	GO:0006032	chitin catabolic process	9/1062	6.36E-05	9
	GO:0016998	cell wall macromolecule catabolic process	9/1062	6.36E-05	9
	GO:0015267	channel activity	8/1062	8.02E-05	8
	GO:0000160	phosphorelay signal transduction system	9/1062	0.000134612	9
	GO:0004857	enzyme inhibitor activity	10/1062	0.000352943	10
	GO:0004497	monooxygenase activity	37/1062	0.000370998	37
	GO:0016491	oxidoreductase activity	31/1062	0.000398176	31
	GO:0004190	aspartic-type endopeptidase activity	12/1062	0.000552306	12
	GO:0022857	transmembrane transporter activity	22/1062	0.000715035	22
	GO:0000981	DNA-binding transcription factor activity, RNA polymerase II-specific	9/1062	0.00092833	9
	GO:0008610	lipid biosynthetic process	6/1062	0.001081111	6
	GO:0008889	glycerophosphodiester phosphodiesterase activity	4/1062	0.001310914	4
	GO:0045490	pectin catabolic process	12/1062	0.001668086	12
	GO:0006071	glycerol metabolic process	4/1062	0.001828939	4
	GO:0102682	N6-(Delta2-isopentenyl)-adenosine 5'-monophosphate phosphoribohydrolase activity	4/1062	0.001828939	4
	GO:0006541	glutamine metabolic process	5/1062	0.003893209	5

GO:0009691	cytokinin biosynthetic process	4/1062	0.004196452	4
GO:0071555	cell wall organization	12/1062	0.004200841	12
GO:0004553	hydrolase activity, hydrolyzing O-glycosyl compounds	16/1062	0.004234197	16
GO:0016747	acyltransferase activity, transferring groups other than amino-acyl groups	16/1062	0.006149076	16
GO:0071949	FAD binding	8/1062	0.006841784	8
GO:0030145	manganese ion binding	10/1062	0.00807502	10
GO:0015074	DNA integration	9/1062	0.008293464	9
GO:0015995	chlorophyll biosynthetic process	4/1062	0.009735098	4
GO:0016705	oxidoreductase activity, acting on paired donors, with incorporation or reduction of molecular oxygen	31/1062	0.009890019	31
GO:0046856	phosphatidylinositol dephosphorylation	5/1062	0.010052386	5
GO:0008061	chitin binding	4/1062	0.011620708	4
GO:0030599	pectinesterase activity	9/1062	0.012980453	9
GO:0042545	cell wall modification	9/1062	0.012980453	9
GO:0045330	aspartyl esterase activity	9/1062	0.012980453	9
GO:0005509	calcium ion binding	23/1062	0.013066826	23
GO:0006535	cysteine biosynthetic process from serine	3/1062	0.013296906	3
GO:0097573	glutathione oxidoreductase activity	6/1062	0.013902354	6
GO:0009664	plant-type cell wall organization	5/1062	0.014801377	5
GO:0019432	triglyceride biosynthetic process	3/1062	0.016737297	3
GO:0042393	histone binding	3/1062	0.016737297	3
GO:0016791	phosphatase activity	7/1062	0.017384442	7
GO:0008171	O-methyltransferase activity	8/1062	0.018175681	8
GO:0043531	ADP binding	30/1062	0.01907781	30
GO:0016787	hydrolase activity	23/1062	0.019316756	23
GO:0042910	xenobiotic transmembrane transporter activity	6/1062	0.020404733	6
GO:0016760	cellulose synthase (UDP-forming) activity	7/1062	0.023760762	7
GO:0003993	acid phosphatase activity	4/1062	0.024479704	4
GO:0006817	phosphate ion transport	3/1062	0.024969858	3
GO:0009736	cytokinin-activated signaling pathway	3/1062	0.024969858	3
GO:0016844	strictosidine synthase activity	3/1062	0.024969858	3
GO:0046873	metal ion transmembrane transporter activity	3/1062	0.024969858	3
GO:0004842	ubiquitin-protein transferase activity	13/1062	0.026415351	13
GO:1990961	xenobiotic detoxification by transmembrane export across the plasma membrane	5/1062	0.028331453	5
GO:0030570	pectate lyase activity	3/1062	0.029763721	3
GO:0030244	cellulose biosynthetic process	7/1062	0.03378126	7
GO:0030246	carbohydrate binding	23/1062	0.037571924	23
GO:0016298	lipase activity	5/1062	0.040652339	5
GO:0000272	polysaccharide catabolic process	3/1062	0.040689583	3
GO:0005249	voltage-gated potassium channel activity	3/1062	0.040689583	3
GO:0030048	actin filament-based movement	3/1062	0.040689583	3
GO:0009055	electron transfer activity	7/1062	0.046276003	7

	GO:0005992	trehalose biosynthetic process	3/1062	0.046807634	3
ZM1 vs. ZM3	GO:0004497	monooxygenase activity	81/1929	1.58E-11	81
	GO:0009523	photosystem II	17/1929	2.86E-11	17
	GO:0009522	photosystem I	15/1929	1.82E-10	15
	GO:0016705	oxidoreductase activity, acting on paired donors, with incorporation or reduction of molecular oxygen	76/1929	4.12E-10	76
	GO:0048046	apoplast	42/1929	7.10E-10	42
	GO:0043531	ADP binding	75/1929	1.63E-09	75
	GO:0009765	photosynthesis, light harvesting	13/1929	2.30E-09	13
	GO:0043565	sequence-specific DNA binding	45/1929	5.94E-09	45
	GO:0004568	chitinase activity	17/1929	6.01E-09	17
	GO:0006032	chitin catabolic process	17/1929	6.01E-09	17
	GO:0016998	cell wall macromolecule catabolic process	17/1929	6.01E-09	17
	GO:0009535	chloroplast thylakoid membrane	22/1929	9.71E-09	22
	GO:0042744	hydrogen peroxide catabolic process	28/1929	2.91E-08	28
	GO:0016168	chlorophyll binding	13/1929	2.99E-08	13
	GO:0005576	extracellular region	43/1929	4.49E-08	43
	GO:0006979	response to oxidative stress	29/1929	1.68E-07	29
	GO:0030145	manganese ion binding	24/1929	2.25E-07	24
	GO:0016788	hydrolase activity, acting on ester bonds	25/1929	9.15E-07	25
	GO:0018298	protein-chromophore linkage	14/1929	1.54E-06	14
	GO:0003777	microtubule motor activity	15/1929	2.35E-06	15
	GO:0016762	xyloglucan:xyloglucosyl transferase activity	12/1929	4.04E-06	12
	GO:0004601	peroxidase activity	28/1929	5.11E-06	28
	GO:0016747	acyltransferase activity, transferring groups other than amino-acyl groups	33/1929	5.91E-06	33
	GO:0071555	cell wall organization	24/1929	8.64E-06	24
	GO:0016760	cellulose synthase (UDP-forming) activity	17/1929	9.62E-06	17
	GO:0007018	microtubule-based movement	17/1929	1.20E-05	17
	GO:0016491	oxidoreductase activity	53/1929	1.74E-05	53
	GO:0030244	cellulose biosynthetic process	17/1929	2.71E-05	17
	GO:0010411	xyloglucan metabolic process	11/1929	3.64E-05	11
	GO:0042546	cell wall biogenesis	11/1929	3.64E-05	11
	GO:0022857	transmembrane transporter activity	37/1929	5.23E-05	37
	GO:0000981	DNA-binding transcription factor activity, RNA polymerase II-specific	14/1929	0.000151153	14
	GO:0008017	microtubule binding	22/1929	0.000156056	22
	GO:0032502	developmental process	7/1929	0.000270498	7
	GO:0005886	plasma membrane	51/1929	0.00046742	51
	GO:0140359	ABC-type transporter activity	21/1929	0.001235646	21
	GO:0015979	photosynthesis	13/1929	0.00145572	13

GO:0016702	oxidoreductase activity, acting on single donors with incorporation of molecular oxygen, incorporation of two atoms of oxygen	7/1929	0.00146906	7
GO:0006633	fatty acid biosynthetic process	16/1929	0.002082114	16
GO:0008171	O-methyltransferase activity	14/1929	0.002519624	14
GO:0005975	carbohydrate metabolic process	52/1929	0.002999523	52
GO:0004089	carbonate dehydratase activity	7/1929	0.00319981	7
GO:0045087	innate immune response	5/1929	0.003286087	5
GO:0004857	enzyme inhibitor activity	12/1929	0.003350313	12
GO:0015074	DNA integration	14/1929	0.004075619	14
GO:0015267	channel activity	8/1929	0.004125023	8
GO:0008061	chitin binding	6/1929	0.004553845	6
GO:0006284	base-excision repair	5/1929	0.004612723	5
GO:0009636	response to toxic substance	4/1929	0.005454018	4
GO:0010158	abaxial cell fate specification	4/1929	0.005454018	4
GO:0010181	FMN binding	7/1929	0.00616905	7

Table S2. KEGG enrichment analyses of DEGs

Group	ID	Description	GeneRatio	pvalue	Count
ZM1 vs. ZM2	ko00940	Phenylpropanoid biosynthesis	51/383	5.26E-18	51
	ko00945	Stilbenoid, diarylheptanoid and gingerol biosynthesis	15/383	3.85E-06	15
	ko00941	Flavonoid biosynthesis	15/383	1.35E-05	15
	ko00073	Cutin, suberine and wax biosynthesis	11/383	2.97E-05	11
	ko00999	Biosynthesis of various plant secondary metabolites	12/383	0.000193205	12
	ko00500	Starch and sucrose metabolism	25/383	0.000201307	25
	ko00950	Isoquinoline alkaloid biosynthesis	13/383	0.000448171	13
	ko00460	Cyanoamino acid metabolism	12/383	0.000966669	12
	ko00350	Tyrosine metabolism	12/383	0.005388332	12
	ko00965	Betalain biosynthesis	5/383	0.006083748	5
	ko04075	Plant hormone signal transduction	29/383	0.006666518	29
	ko00360	Phenylalanine metabolism	9/383	0.010939736	9
	ko04016	MAPK signaling pathway - plant	19/383	0.01124935	19
	ko02010	ABC transporters	9/383	0.01570766	9
	ko00901	Indole alkaloid biosynthesis	4/383	0.018440347	4
	ko00100	Steroid biosynthesis	6/383	0.021442164	6
	ko00910	Nitrogen metabolism	8/383	0.023915786	8
	ko00196	Photosynthesis - antenna proteins	4/383	0.035422199	4
	ko00603	Glycosphingolipid biosynthesis - globo and isoglobo series	3/383	0.03879211	3
	ko00740	Riboflavin metabolism	4/383	0.046428443	4
ZM2 vs. ZM3	ko00940	Phenylpropanoid biosynthesis	40/327	6.10E-13	40

	ko00062	Fatty acid elongation	10/327	6.34E-05	10
	ko04075	Plant hormone signal transduction	30/327	0.000281578	30
	ko00908	Zeatin biosynthesis	8/327	0.000773855	8
	ko02010	ABC transporters	9/327	0.005883913	9
	ko00945	Stilbenoid, diarylheptanoid and gingerol biosynthesis	8/327	0.015432952	8
	ko00430	Taurine and hypotaurine metabolism	4/327	0.015461566	4
	ko00592	alpha-Linolenic acid metabolism	8/327	0.016733444	8
	ko04016	MAPK signaling pathway - plant	16/327	0.021737518	16
	ko00591	Linoleic acid metabolism	4/327	0.032120042	4
	ko00750	Vitamin B6 metabolism	3/327	0.037018818	3
ZM1 vs. ZM3	ko00940	Phenylpropanoid biosynthesis	48/563	1.24E-09	48
	ko00196	Photosynthesis - antenna proteins	13/563	3.19E-09	13
	ko00945	Stilbenoid, diarylheptanoid and gingerol biosynthesis	19/563	1.42E-06	19
	ko00941	Flavonoid biosynthesis	17/563	9.56E-05	17
	ko04075	Plant hormone signal transduction	43/563	0.000824647	43
	ko04016	MAPK signaling pathway - plant	29/563	0.001000002	29
	ko00592	alpha-Linolenic acid metabolism	14/563	0.001277381	14
	ko00950	Isoquinoline alkaloid biosynthesis	15/563	0.001964772	15
	ko00520	Amino sugar and nucleotide sugar metabolism	27/563	0.002884056	27
	ko00073	Cutin, suberine and wax biosynthesis	10/563	0.003262664	10
	ko00591	Linoleic acid metabolism	7/563	0.003799245	7
	ko00195	Photosynthesis	13/563	0.007690849	13
	ko00966	Glucosinolate biosynthesis	5/563	0.009492058	5
	ko00053	Ascorbate and aldarate metabolism	12/563	0.011248271	12
	ko00910	Nitrogen metabolism	11/563	0.013198894	11
	ko00903	Limonene and pinene degradation	4/563	0.017101761	4
	ko00561	Glycerolipid metabolism	15/563	0.022067494	15
	ko00730	Thiamine metabolism	6/563	0.026674047	6
	ko02010	ABC transporters	11/563	0.027218077	11
	ko00400	Phenylalanine, tyrosine and tryptophan biosynthesis	10/563	0.042968782	10
	ko00460	Cyanoamino acid metabolism	11/563	0.045898361	11
	ko00480	Glutathione metabolism	20/563	0.048113632	20

Table S3. Degree values of proteins in the PPI network.

Protein	Degree
AUX1	20
GH3.6	10
PP2CA	10
IAA26	9
IAA4	9
GH3.1	9
EIN3	9
IAA27	8

ARF5	8
ARR9	7
ARF3	6
GH3.9	6
ARR3	5
PIF3	5
RGL3	5
IAA12	4
PR1	4
ERF1B	4
PIF4	4
IAA30	3
SAUR36	3
PYL2	1
PYL8	1

Table S4. List of primers

Sequence ID	Unigene ID	Primer ID	Primer Sequence
CcIAA30	*Cca.gene36235*	CcIAA30-F	CCTTCACCAGCGGTAACTGT
		CcAIAA30-R	CCAATGGCCTCCGATCCTTT
CcARF5	*Cca.gene31872*	CcARF5-F	GGCAACTTGATGACCCCAGA
		CcARF5-R	AGTCGTTGGCATCTTCAGGG
CcGH3.9	*Cca.gene23794*	CcGH3.9-F	CTCATTTCTGCCCACCCCAT
		CcGH3.9-R	GGTTGACTTCTCCGCCTTGA
CcSAUR	*Cca.gene3046*	CcSAUR-F	GCTGTCTATGTGGGCGATGA
		CcSAUR-R	AGTTCCTCCATCTCCAGGCT
CcAHP	*Cca.gene8703*	CcAHP-F	TCACCAGTTGAAAGGCAGCA
		CcAHP-R	CCTGCTGCCAGAATTTGCTG
CcB-ARR	*Cca.gene29885*	CcB-ARR-F	TCCCAGAGATCAGCGAGGAA
		CcB-ARR-R	CTCCAAGGCTGCTTCTGTGA
CcA-ARR	*Cca.gene26962*	CcA-ARR-F	CCTTGACACTGGAGCAGAGG
		CcA-ARR-R	TGCAACTGTGACTCCAGCAA
CcDELLA	*Cca.gene13820*	CcDELLA-F	ATGACGTGTGCAGATTCCGT
		CcDELLA-R	GAGGATCTCATTCTCGGCGG
CcPIF3	*Cca.gene6753*	CcPIF3-F	TCACCAATCGCATTGTGGGA
		CcPIF3-R	CGAGTTACCTGCAGCTGACA
CcPYL8	*Cca.gene19502*	CcPYL8 -F	AGTGCAGTTCAGCTCTCGTC
		CcPYL8-R	GTGCTTGTTGTTGCTGGGAG
CcLHT8	*Cca.gene34527*	CcLHT8-F	CCAAGCTCGACCCACAAGAT
		CcLHT8-R	TGGAGCTGAGTCAGGATCCA
CcPOD	*Cca.gene29568*	CcPOD-F	ATTCTTCTCTCGACGCAGCC
		CcPOD-R	TCACCAACTCCATTGTCGCT
CcFAR	*Cca.gene694*	CcFAR-F	ACCATGGCCTCCTTCCAAAG
		CcFAR-R	TTGTCGTCCTCAAGTGCTCC

CcPPO	*Cca.gene34575*	CcPPO-F	CAAGATCCCACCAACACCGA
		CcPPO-R	CGGTCACCGGTCCAGATATG
CcPP2C37	*Cca.gene37353*	CcPP2C37-F	AGATCGTCGTTGGCAACTGT
		CcPP2C37-R	CGGTATCACGTACGGCTTCA
CcERF1	*Cca.gene32090*	CcERF1-F	GAAACGTCCTCAGAGGCCAA
		CcERF1-R	CCACGTATAGCAAAAGCGGC
